# Conserved RNA binding activity of a Yin-Yang 1 homologue in the ova of the purple sea urchin *Strongylocentrotus purpuratus*

**DOI:** 10.1038/s41598-018-26264-0

**Published:** 2018-05-23

**Authors:** Zachery R. Belak, Nicholas Ovsenek, Christopher H. Eskiw

**Affiliations:** 1 Food and Bioproduct Sciences, University of Saskatchewan, Saskatoon, Canada; 2 Anatomy and Cell Biology, University of Saskatchewan, Saskatoon, Canada; 3 Biochemistry, University of Saskatchewan, Saskatoon, Canada

## Abstract

Yin-Yang 1 (YY1) is a highly conserved transcription factor possessing RNA-binding activity. A putative YY1 homologue was previously identified in the developmental model organism *Strongylocentrotus purpuratus* (the purple sea urchin) by genomic sequencing. We identified a high degree of sequence similarity with YY1 homologues of vertebrate origin which shared 100% protein sequence identity over the DNA- and RNA-binding zinc-finger region with high similarity in the N-terminal transcriptional activation domain. SpYY1 demonstrated identical DNA- and RNA-binding characteristics between *Xenopus laevis* and *S. purpuratus* indicating that it maintains similar functional and biochemical properties across widely divergent deuterostome species. SpYY1 binds to the consensus YY1 DNA element, and also to U-rich RNA sequences. Although we detected SpYY1 RNA-binding activity in ova lysates and observed cytoplasmic localization, SpYY1 was not associated with maternal mRNA in ova. SpYY1 expressed in *Xenopus* oocytes was excluded from the nucleus and associated with maternally expressed cytoplasmic mRNA molecules. These data demonstrate the existence of an YY1 homologue in *S. purpuratus* with similar structural and biochemical features to those of the well-studied vertebrate YY1; however, the data reveal major differences in the biological role of YY1 in the regulation of maternally expressed mRNA in the two species.

## Introduction

Yin-Yang 1 (YY1) is a member of the GLI-Kruppel family of transcription factors with activity in activation, repression, or initiation of transcription at numerous cellular and viral promoters depending on the cellular context^1–6^. The activity of YY1 is modified by numerous interactions with other proteins and by extensive posttranslational modifications^7–19^. Regulation of the transcriptional activity of YY1 is also achieved through nucleo-cytoplasmic redistribution of the protein^20–29^. Human YY1 has been most extensively studied as a transcription factor in the context of cancer, and its role therein has been extensively reviewed^1,5,25,30,31^. Recently, YY1 has been implicated as a structural regulator of enhancer-promoter interactions and in mediating long-range DNA interactions^32,33^. The important nature of these many functions of YY1 underlines the need for further understanding of the biochemistry and function of YY1. Indeed, the importance of the YY1 protein is further demonstrated by its high conservation among divergent vertebrate and invertebrate species. In fact, all described vertebrate and invertebrate YY1 homologues consist of four C2H2-type zinc-finger domains occupying the C-terminal portion of the protein and responsible for DNA-binding activity, an N-terminal bipartite transcriptional activation domain, and a transcriptional repression domain near the C-terminus^1,34–36^.

Although there is a growing pool of information on YY1 function, the role of this protein in embryonic development remains poorly understood. Donohoe *et al*. studied YY1 knockout mice and established an essential role for YY1 in vertebrate development^37^. Homozygous YY1 knockout embryos exhibited peri-implantation lethality while heterozygous embryos survived implantation but went on to display a number of defects such as lowered implantation efficiency, neurulation defects, and exencephaly^37^. Analysis of the nucleo-cytoplasmic distribution of YY1 in mouse development revealed it is entirely cytoplasmic in oocytes and zygotes until the 2-cell stage and present in both nuclear and cytoplasmic compartments of cells of the blastocyst, inner cell mass, and trophoectoderm in stage E3.5 embryos^37^. YY1 has been shown to be critical for neurological development in *Xenopus laevis*^38^ and has been implicated as a regulator of gene expression downstream of transforming growth factor beta (TGF-β) and bone morphogenic protein (BMP) signalling via interaction with MAD and Smad proteins in a developmental context^39^. Initial studies on YY1 in early development of *Xenopus laevis* were previously performed in our laboratory with the aim of elucidating factors involved in histone gene expression in early development^40–42^. Analysis of YY1 DNA-binding activity through development revealed that while YY1 protein levels remain relatively constant through development, YY1 DNA-binding activity is present only in immature oocytes and in embryos after the mid-blastula transition (MBT)^40,42^. Analysis of the nucleocytoplasmic distribution of YY1 in oocytes and embryos subsequently revealed that it is entirely cytoplasmic from oocyte stage III, through fertilization and MBT, and in the embryo until at least neurulation (embryonic stage 13) suggesting the absence of a transcriptional role during early development^40^. Our previous biochemical analysis of oocytes and embryos has shown that in *Xenopus* YY1 is a component of cytoplasmic RNA-storage particles termed messenger ribonucleoprotein particles (mRNPs)^42^. Presence of YY1 in mRNPs was confirmed by oligo-dT cellulose chromatography of oocyte lysates, with retention of YY1 on oligo-dT cellulose matrix dependent on presence of intact polyadenylated mRNA^42–44^. The association of YY1 with mRNPs is dependent on YY1 RNA-binding activity. This was demonstrated *in vitro* using recombinant *Xenopus* YY1 protein, and confirmed with native YY1 purified from oocytes and by experiments demonstrating that microinjected RNA substrates block association of YY1 with mRNA *in vivo*^43^. YY1 has high affinity for RNA, with a similar magnitude of affinity for DNA substrates^43,44^. Biochemical experiments show YY1-RNA interactions are highly stable both *in vitro* and *in vivo*^43,44^. Our analysis of YY1 RNA-binding activity using *X. laevis* YY1 indicated that YY1 can bind to single and double-stranded U-rich RNA, and indeed, our laboratory was the first to describe the now well-known RNA binding activity of YY1^43,44^. The recent and very thorough analysis of human YY1 by Wai *et al*. supports these findings, although it appears human YY1 binds to single stranded RNA only^45^. Recently, the RNA-binding activity of YY1 has been implicated in a number of biological processes. Evidence indicates that binding of YY1 to non-coding RNA is important for tethering of YY1 to enhancer sites^46^, and binding of YY1 to the *Xist* RNA is thought to underpin recruitment of the *Xist* RNA to the X-chromosome during X-inactivation^47^.

Fung *et al*. have identified *cis-*regulatory elements of the *SpFoxB* gene in the purple sea urchin *Strongylocentrotus purpuratus* with sequences identical to YY1 *cis-*regulatory elements found in higher animals^48^. It was demonstrated that a specific protein-nucleic acid binding activity with high affinity for the YY1 DNA consensus site exists in the *S. purpuratus* embryo and that the putative YY1-binding site found in the *SpFoxB* gene is able to modulate transcription in reporter assays^48^. Similarly, a putative YY1 consensus binding site has also been identified in the promotor of the *Hbox12* gene of the sea urchin *Paracentrotus lividus* and its deletion affects the spatial, temporal, and magnitude of expression of *Hbox12*-promoter driven GFP fusion reporter constructs^49^. These analysis demonstrated transcriptional activity from the YY1 consensus binding site in fertilized eggs and early morula-stage embryos, implying that YY1 functions as a nuclear transcription factor early in development, though the specific stage at which YY1-modulated transcription begins in the ovum or embryo is unknown^48–50^. The *Strongylocentrotus purpuratus* genome sequence (genomic contig. NW_011983912.1) predicts a 1,203 bp open reading frame (XM_785095.4) encoding a 400 amino acid polypeptide (XP_790188) with Mr = 44.6 kDa and high sequence similarity to the vertebrate nucleic acid binding protein YY1. These data strongly indicates the presence of a functional YY1 homologue in *S. purpuratus*, although to date no such protein has been cloned or characterized. Gustafson and Wessel termed the putative *S. purpuratus* YY1 homologue *Sp-Pho* based on its classification as a member of the polycomb group of proteins and similarity to *Drosophila melanogaster PHO* and used quantitative PCR and *in situ* hybridization to analyze the expression of its mRNA product during embryonic development of the sea urchin^50^. Data demonstrate that *Sp-Pho* mRNA is expressed in the ovum and that mRNA levels decrease overall during embryonic development. *In situ* hybridization showed that *Sp-Pho* transcripts were evenly distributed throughout the early embryo with slight enrichment in the vegetal plate, primary mesenchyme, and oral ectoderm of later embryos^50^. To date, no studies involving knock-down or knock-out of YY1 in the sea urchin have been reported, underlining the current lack of data on the role of YY1 in sea urchin development. In the current study, we describe the cloning of the *S. purpuratus* YY1 homologue, the characterization of its nucleic-acid binding activity, and an analysis of its subcellular localization. Furthermore, we suggest the use of the name *SpYY1* for this gene and its protein product since our analysis described below shows far closer sequence homology of the sea urchin protein to mammalian YY1 than to the *Drosophila melanogaster* YY1 homologue *PHO*.

## Results

### Sequence analysis of SpYY1

Previous observations of an unidentified nucleic acid-binding activity with affinity for the consensus YY1 DNA-binding element suggested that SpYY1 is expressed in embryos of *S. purpuratus*^48^. Furthermore, the *S. purpuratus* genomic sequence contains an open reading frame encoding a protein highly similar to vertebrate YY1^48^. Initial Western blots of *S. purpuratus* ova lysate probed with anti-Xenopus YY1 antibodies revealed a single ~47 kDa band consistent with the predicted size of SpYY1 based on genomic sequencing (data not shown). We began our analysis of *S. purpuratus* YY1 by amplifying the coding sequence of the predicted mRNA in order to further characterize the encoded protein. We prepared primers flanking the predicted coding sequence and employed them in PCR reactions containing *S. purpuratus* ovum cDNA. These reactions resulted in amplification of a fragment of 1.2 kb as expected from the genomic sequence (data not shown). Following digestion and cloning into pRsetB, the fragment was sequenced and found to be identical to the sequence predicted in XM_785095.4 by genomic sequencing. These data indicate that the predicted *S. purpuratus* YY1 (SpYY1) mRNA sequence is indeed expressed in ova, encodes a 44.6 kDa polypeptide, and that the polypeptide is cross-reactive with antibodies raised against epitopes specific to YY1.

**Figure 1:**
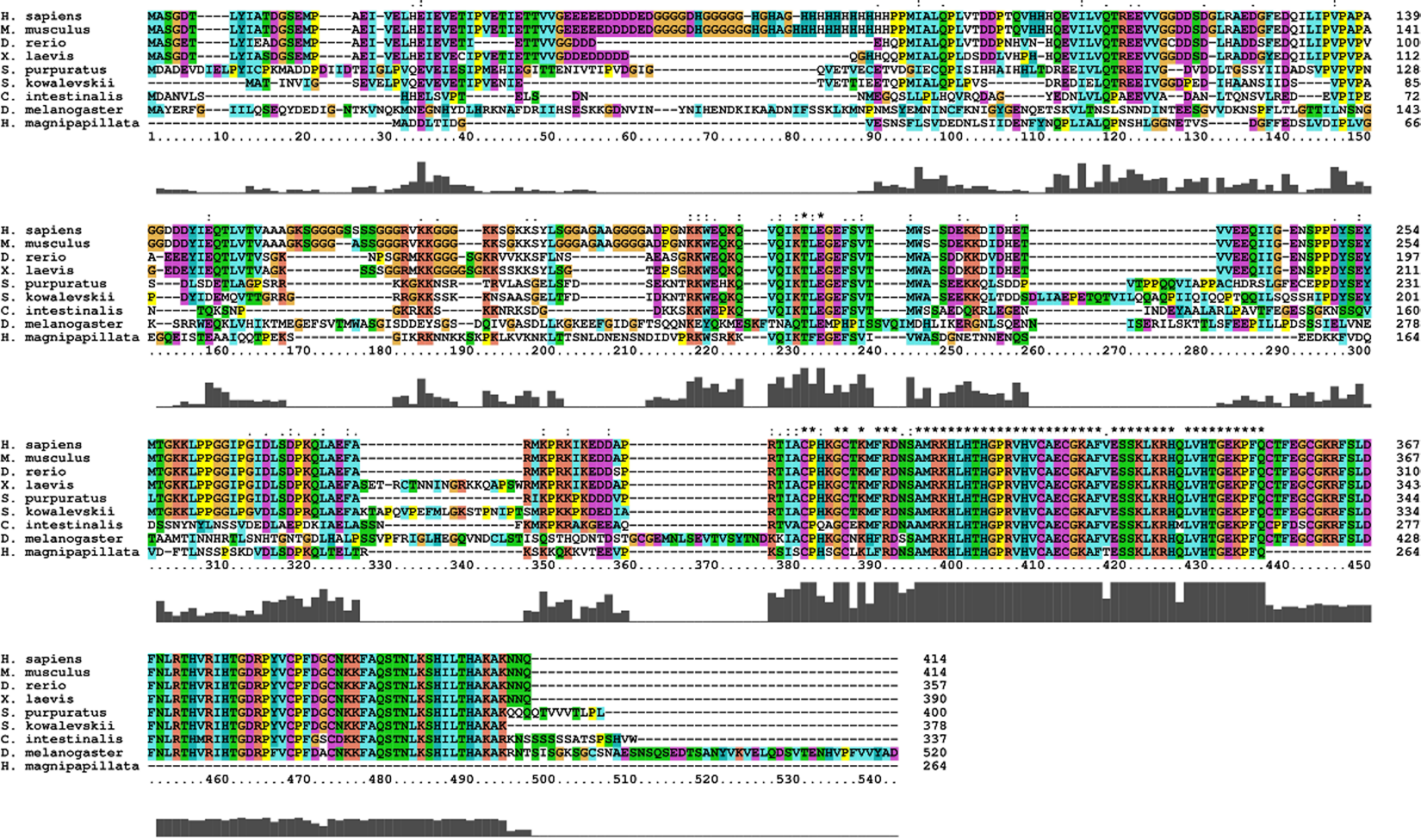
Multiple Sequence Alignment of SpYY1. Results of multiple sequence alignment of YY1 homologues from a diverse set of species using ClustalX version 2.1 software.

Following cloning and sequencing of *SpYY1*, we then applied computational analysis to compare the sequence to that of YY1 homologues from a variety of species (Fig. 1). Clustal analysis revealed that SpYY1 is highly similar to YY1 homologues from both deuterostome and protostome animals. Remarkably, the regions spanning consensus sequence residues 229–241, 298–328, 351–359, and 379–499 were nearly identical between the deuterostomes *H. sapiens, M. musculus, D. rerio, X. laevis*, and *S. purpuratus*; and were highly similar in the deuterostome *C. intestinalis* (Fig. 1). The region spanning 229–241 of the consensus sequence was also moderately conserved between deuterostome and protostome animals. The region spanning consensus sequence residues 379–499 was highly conserved between the deuterostomes, the protostome *D. melanogaster*, and the cnidarian *H. magnapapillata*. Highly conserved regions around residues 229–241 of the consensus sequence correlate with regions of mammalian YY1 known to mediate transcriptional activation^34^. The highly conserved C-terminal region corresponds to the zinc-finger region of the protein responsible for interaction with DNA and RNA also in addition to a bipartite transcriptional repression domain previously characterized in mammalian YY1^34,35,43–45^. These observations indicate that YY1 in *Strongylocentrotus purpuratus*, and in other metazoans, is likely to exhibit similar biochemical properties and functions to those previously identified in vertebrates.

**Figure 2:**
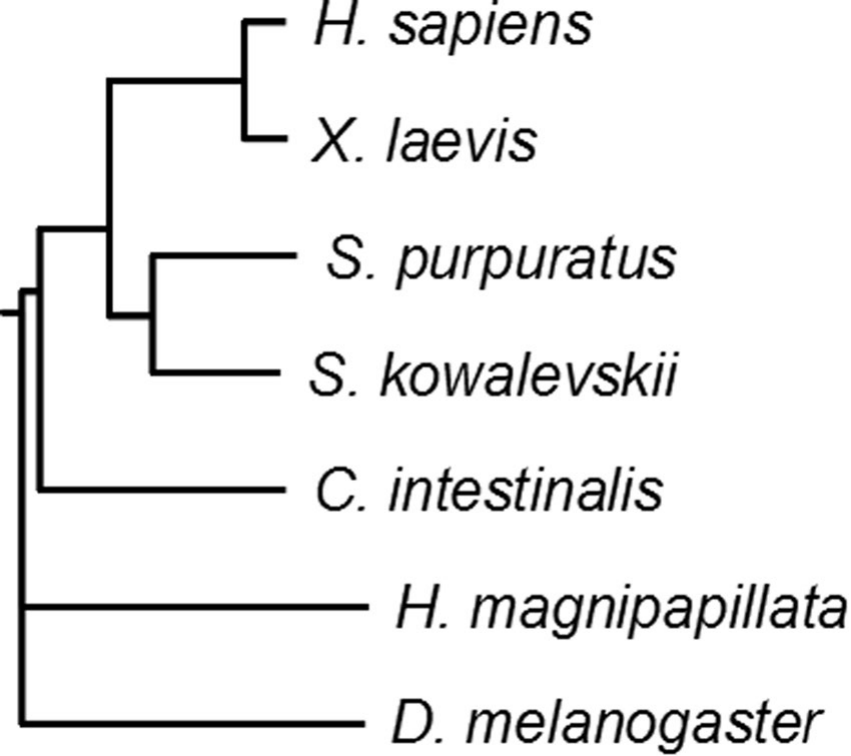
Phylogenetic Comparison of YY1 from Assorted Metazoan Species. A phylogenetic tree comparing YY1 homologues from a diverse set of species was constructed using the drawgram program from the PHYLIP version 3.69 software package using the default parameters.

Phylogenetic analysis of multiple sequence alignment data was used to generate the phylogenetic tree in Fig. 2. As expected, *H. sapiens* and *X. laevis* sequences were more closely related phylogenetically to each other than to those of other species. The *S. purpuratus* sequence was found to be most similar to that of the hemichordate *S. kowalevskii*, consistent with the accepted view that hemichordates and echinoderms form an evolutionary clade (Fig. 2)^51,52^. Overall, the sequence alignment is consistent with the accepted evolutionary relationships of the organisms studied, and demonstrates visually that the gene identified is likely to be functionally and biochemically the *S. purpuratus* equivalent to YY1 in vertebrates and hemichordates.

### Expression of Strongylocentrotus purpuratus YY1 *In Vitro*

**Figure 3:**
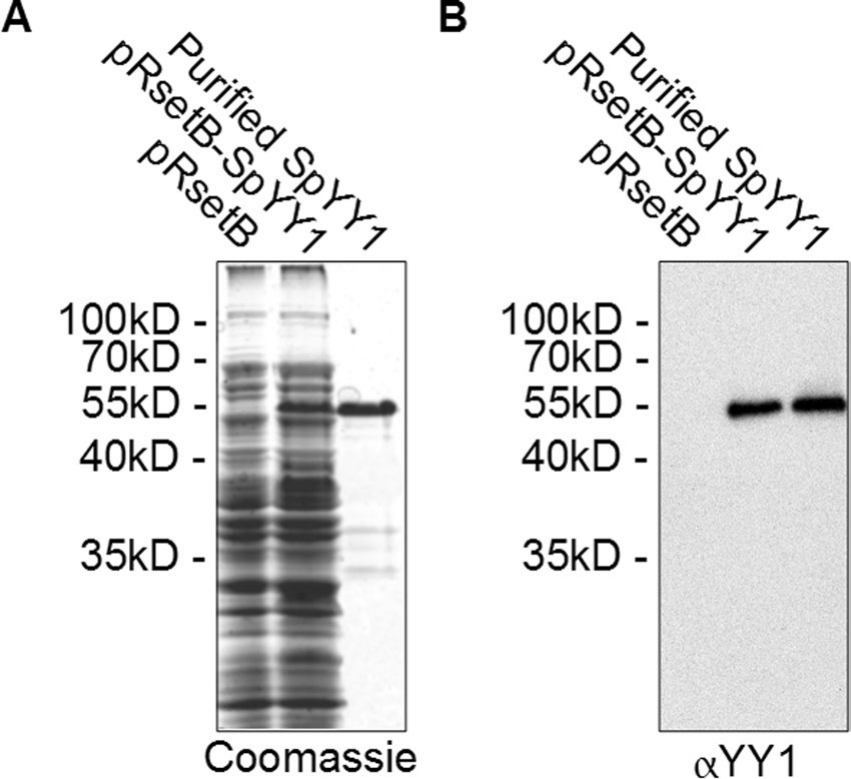
Expression of *Strongylocentrotus purpuratus* YY1 in *E. coli*. The *S. purpuratus* YY1 coding sequence was cloned into the bacterial expression vector pRsetB to yield pRSetB-SpYY1. Both empty vector (pRsetB) and the construct pRsetB-SpYY1 were then transformed into BL21(DE3)pLysS *E. coli* and expression of exogenous protein was induced by addition of IPTG. The expressed SpYY1 protein was then purified using immobilized metal affinity chromatography according to previously described procedures. Bacterial lysates (pRsetB, pRsetB-SpYY1; above each panel) as well as the purified protein preparation (Purified SpYY1; above each panel) were analyzed by SDS-PAGE and Western blotting. (**A**) SDS-PAGE gel stained with Coomassie brilliant blue. (**B**) Western blot of bacterial lysates and purified protein probed with anti-*Xenopus* YY1 antibody. Positions of molecular mass markers are indicated to the left of each panel.

Transformation and expression of the SpYY1 construct described above into BL21(DE3) pLysS *E. coli* resulted in synthesis of the expected ~47 kDa polypeptide (the C-terminal His-tag and EK cleavage sites of pRSetB add approximately 5 kDa to the apparent mass of polypeptides expressed from this vector) (Fig. 3). The expressed protein was immunoreactive to anti-HIS antibody (data not shown), verifying its origin by expression from the transformed pRsetB vector. It was also recognized by anti-YY1 antibody (Fig. 3B). In order to further characterize the biochemical properties of SpYY1 we bacterially expressed the protein and carried out purification via immobilized metal affinity chromatography followed by size exclusion chromatography using methods developed in our lab for the production of zinc finger proteins (previously described in^53^). The resulting highly purified protein preparation (Fig. 3A) was subsequently utilized in experiments analyzing the nucleic acid-binding activity of SpYY1.

### DNA and RNA-binding activity of SpYY1

**Figure 4:**
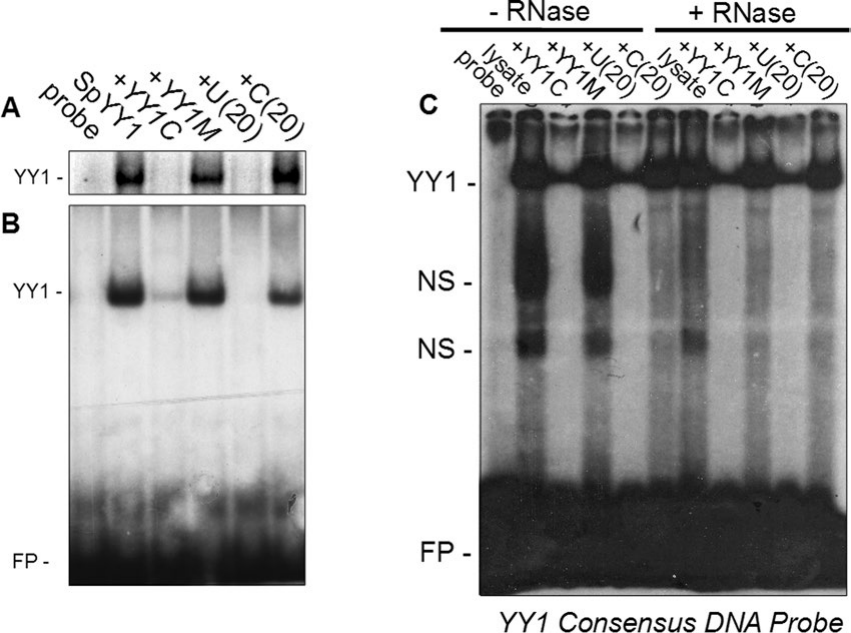
DNA and RNA binding activity of recombinant SpYY1 and SpYY1 in *S. purpuratus* ovum lysates. Bacterially expressed and purified *S. purpuratus* YY1 (250 nM) was combined with 10 nM radiolabelled YY1 DNA consensus probe (A, upper panel) or 10 nM end-labelled U(20) RNA probe (B, lower panel) and various competitor oligonucleotides as indicated, and analyzed by EMSA. For analysis of DNA binding activity in ova, either untreated (−RNase) or RNase treated (+RNase) lysates of *S. purpuratus* ova were analyzed in EMSA reactions containing 10 nM radiolabelled YY1 DNA consensus probe and various competitor oligonucleotides as indicated above the panel (C). Competitors are indicated above the panel: SpYY1, YY1 protein only, no competitor; +YY1C, 100 nM (10X excess) unlabelled YY1 DNA consensus oligonucleotide; +YY1M, 100 nM (10X excess) unlabelled YY1 DNA mutant consensus oligonucleotide; +U(20), 100 nM (10X excess) unlabelled U(20) RNA; +C(20), 100 nM (10X excess) unlabelled C(20) RNA. The positions of YY1/DNA and YY1/RNA complexes and unbound RNA oligonucleotide (free probe, FP) are indicated to the left of the panels.

YY1 interacts with its cognate DNA sequence via the four C-terminal zinc-finger domains^35^, and has high affinity for RNA substrates^43–45^. The zinc fingers of YY1 have also been shown to be responsible for RNA-binding^45^. Given that the amino acid sequence of SpYY1 is identical to the human and *Xenopus* homologues over the region containing the zinc-fingers (Fig. 1), we hypothesized that SpYY1 would have nearly identical nucleic acid-binding properties as YY1 of vertebrate origin. In order to compare the respective nucleic acid binding activities, highly purified recombinant SpYY1 was analyzed by electrophoretic mobility shift assays (EMSA) using radiolabeled DNA and RNA probes for which vertebrate YY1 has high affinity^43,44^. SpYY1 bound to DNA containing the YY1 consensus sequence (Fig. 4A) and this binding activity was specifically competed by a 10-fold excess of unlabeled consensus DNA, but was not competed by a DNA oligonucleotide competitor containing a CCGGCCGCGATT base substitution at the YY1 binding site. The SpYY1-DNA complex was also competed efficiently by a 10-fold excess of single-stranded poly-U RNA, a previously identified high-affinity substrate of vertebrate YY1, but not by poly-C RNA for which vertebrate YY1 has low affinity^43^. The ability of U(20) RNA to effectively compete SpYY1-DNA interactions implies the existence of an RNA-binding activity in SpYY1, as has been observed in the *Xenopus* homologue. We directly tested the ability of SpYY1 to bind RNA by EMSA analysis using recombinant SpYY1 protein and radiolabeled U20 RNA probe. The data presented in Fig. 4B show that SpYY1 is able to bind directly to U(20) RNA probe. Furthermore, the SpYY1-RNA interaction was specifically competed by YY1 consensus DNA oligonucleotide but not by the mutant YY1 DNA oligonucleotide. SpYY1-RNA complexes were also competed by tenfold excess of unlabeled U(20) RNA but not by C(20) RNA, consistent with the observed affinity of *Xenopus* YY1 for U-rich RNA substrates^43^. Importantly, these data indicate that DNA and RNA-binding are mutually exclusive in SpYY1, as has been previously observed for vertebrate YY1. Since the DNA-binding activity of YY1 is localized to the zinc-finger domains of the protein, the displacement of DNA substrates by RNA in binding reactions supports the observation that RNA-binding activity resides in the zinc-finger region^45^.

### YY1 DNA-binding and RNA-binding activity in *S. purpuratus* ova lysates

**Figure 5:**
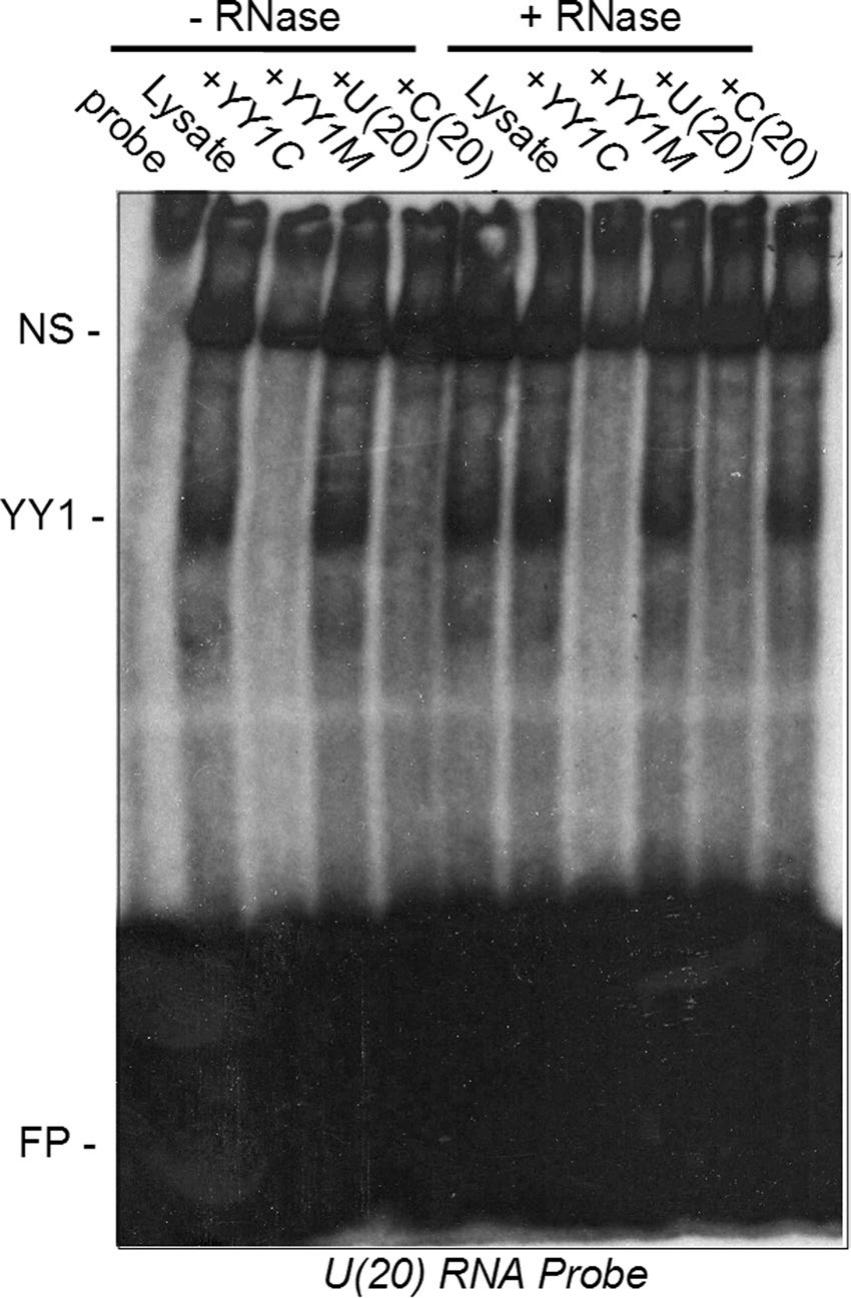
RNA binding activity of YY1 in *S. purpuratus* ovum lysates. Either untreated (−RNase) or RNase treated (+RNase) lysates of *S. purpuratus* ova were analyzed in EMSA reactions containing 10 nM radiolabelled poly-U(20) RNA probe and various competitor oligonucleotides as indicated above the panel. Competitors were: +YY1C, 100 nM (10X excess) unlabelled YY1 DNA consensus oligonucleotide; +YY1M, 100 nM (10X excess) unlabelled YY1 DNA mutant consensus oligonucleotide; +U(20), 100 nM (10X excess) unlabelled U(20) RNA; +C(20), 100 nM (10X excess) unlabelled C(20) RNA. The positions of the YY1/RNA (YY1) and non-specific (NS) complexes, as well as free probe (FP) are indicated to the left of the panel.

Having observed strong DNA and RNA-binding activity of recombinant SpYY1, we next examined the presence of YY1 nucleic acid-binding activity in ova lysates using EMSA (Figs 4C and 5). While YY1 is abundant in *Xenopus* oocytes, no YY1 DNA-binding activity is detectable since all cellular YY1 is present in complex with RNA, however treatment of oocyte lysates with RNase results in unmasking of YY1 DNA-binding activity^40,42,43^. Binding reactions containing 0.1 pmol radiolabelled YY1 consensus DNA oligonucleotide revealed a relatively strong YY1 DNA-binding activity in *S. purpuratus* ova which was competed specifically by a tenfold excess of unlabelled consensus DNA element or U(20) RNA (Fig. 4C). This binding activity was not competed by either mutant YY1 consensus DNA oligonucleotide or C(20) RNA. In *Xenopus* oocytes, both the DNA- and RNA-binding activities of YY1 are more readily detectible after RNase treatment of lysates since YY1 is tightly bound into mRNP complexes^40,42,43^. However, in sea urchin eggs, pre-treatment of lysates with RNase A/T1 mixture did not result in a significant change in the quantity of YY1 DNA-binding activity (Fig. 4C).

We next analyzed lysates for YY1 RNA-binding activity with EMSA using a radiolabelled U(20) RNA probe for which *Xenopus* has high affinity^43,44^. A specific YY1 RNA-binding activity was found in lysates which was competed efficiently by excess YY1 consensus DNA oligonucleotide and by unlabelled U(20) RNA but not by either YY1 mutant consensus DNA oligonucleotide or by C(20) RNA (Fig. 5). Similar to the results obtained using DNA probes, no difference in the intensity of YY1 RNA-binding activity was observed following RNase treatment of lysates prior to analysis by EMSA. The observations of abundant RNA and DNA binding activity in untreated lysates (Figs 4C and 5) strongly suggest that most, if not all, YY1 is free and unbound to RNA in the ova of this species.

### Association of SpYY1 with polyadenylated mRNA in ova of *S. purpuratus*

**Figure 6:**
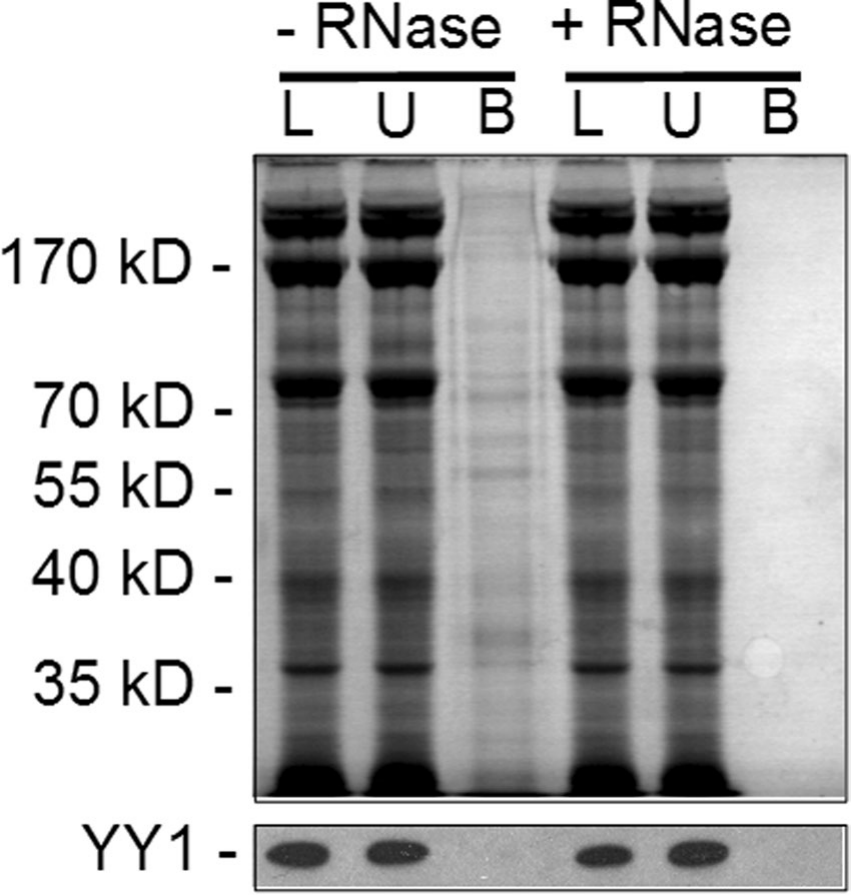
Isolation of Messenger Ribonucleoprotein Particles from *S. purpuratus* ova. Lysates of *S. purpuratus* ova were either untreated (−RNase) or treated with RNase A/T1 (+RNase) and applied to oligo-dT cellulose columns and the bound proteins analyzed by SDS-PAGE and visualized by staining with Coomassie brilliant blue (upper panel) or subjected to Western blotting with anti-*Xenopus* YY1 antibody (lower panel). Fractions are indicated above the panel, L, load-on; U, unbound fraction; B, bound fraction. Positions of molecular mass markers and the YY1 band found upon Western blotting are indicated to the left of the panel.

In order to examine more directly if SpYY1 associates with polyadenylated mRNA in sea urchin ova, as has been previously shown in oocytes and ova of *Xenopus*, we analyzed extracts by oligo-dT cellulose chromatography (Fig. 6). Oligo-dT cellulose chromatography has a substantial history in the literature as a means of isolating mRNA, and importantly in this case, proteins associated with mRNA in mRNP complexes^42,43^. Analysis of material bound to oligo-dT by Coomassie blue staining of SDS-PAGE gels shows that a number of polypeptides were isolated on oligo-dT matrix in an RNA-dependent manner (Fig. 6, upper panel, compare lanes 3 and 6). These data demonstrate that we were able to efficiently isolate mRNPs from ova lysates of *S. purpuratus*. Analysis of bound and unbound fractions with anti-YY1 antibodies revealed that the entire cellular complement of YY1 was present in the unbound fraction with no detectible YY1 retained on oligo-dT cellulose matrix (Fig. 6, lower panel). These data indicate that YY1 does not appreciably associate with polyadenylated mRNA molecules in the ova of *S. purpuratus*, in contrast to the previously reported role of YY1 as an abundant component of mRNP complexes in *Xenopus*^42,43^.

### Subcellular localization of SpYY1

**Figure 7:**
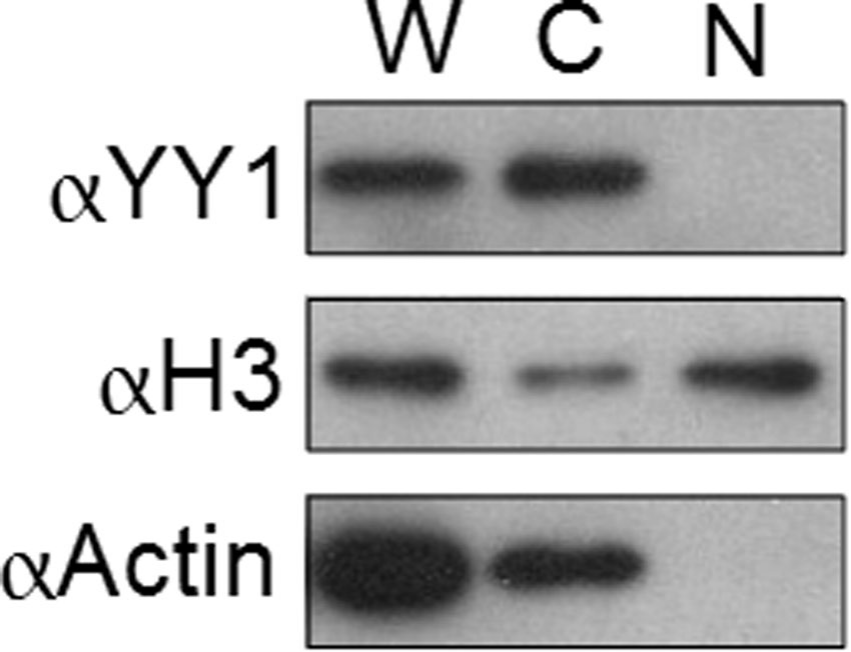
Nucleocytoplasmic Distribution of SpYY1 in *S. purpuratus* ova. Ova of *S. purpuratus* were fractionated into nuclear and cytoplasmic extracts using previously described methods. Whole-cell (W) as well as nuclear (N) and cytoplasmic (C) fractions were analyzed by SDS-PAGE followed by Western blotting. Antibodies used are indicated to the left of the panel, αYY1, Rabbit anti-*Xenopus* YY1; αH3, Goat anti-Histone H3 antibody, nuclear marker; αActin, Goat anti-Beta-Actin antibody, cytoplasmic marker.

We initially sought to examine the subcellular localization of YY1 in *S. purpuratus* ova by immunohistochemistry. However, despite considerable effort and attempts at using various combinations of fixative and antigen recovery, we were unable to detect YY1 in ova sections using the antibody described above (data not shown). Therefore, we prepared nuclear and cytoplasmic fractions of ova and analyzed them for the presence of YY1 by Western blotting (Fig. 7). YY1 was detected only in the cytoplasmic fraction. As controls, the same samples were analyzed by using anti-β-actin and anti-histone H3 antibodies. Blots probed with anti-histone H3 antibody revealed that the majority of the signal was found in the nuclear fraction as expected, with some nuclear contamination of the cytoplasmic fraction (Fig. 7). Application of anti-β-actin antibodies revealed that β-actin was found exclusively in the cytoplasmic fraction and demonstrated that the nuclear fraction was uncontaminated by cytoplasmic material. Taken together, these data show that YY1 was confined to the cytoplasm and is excluded from the nucleus in *S. purpuratus* ova.

### Messenger RNA interactions and nuclear exclusion of SpYY1 expressed in Xenopus oocytes

**Figure 8:**
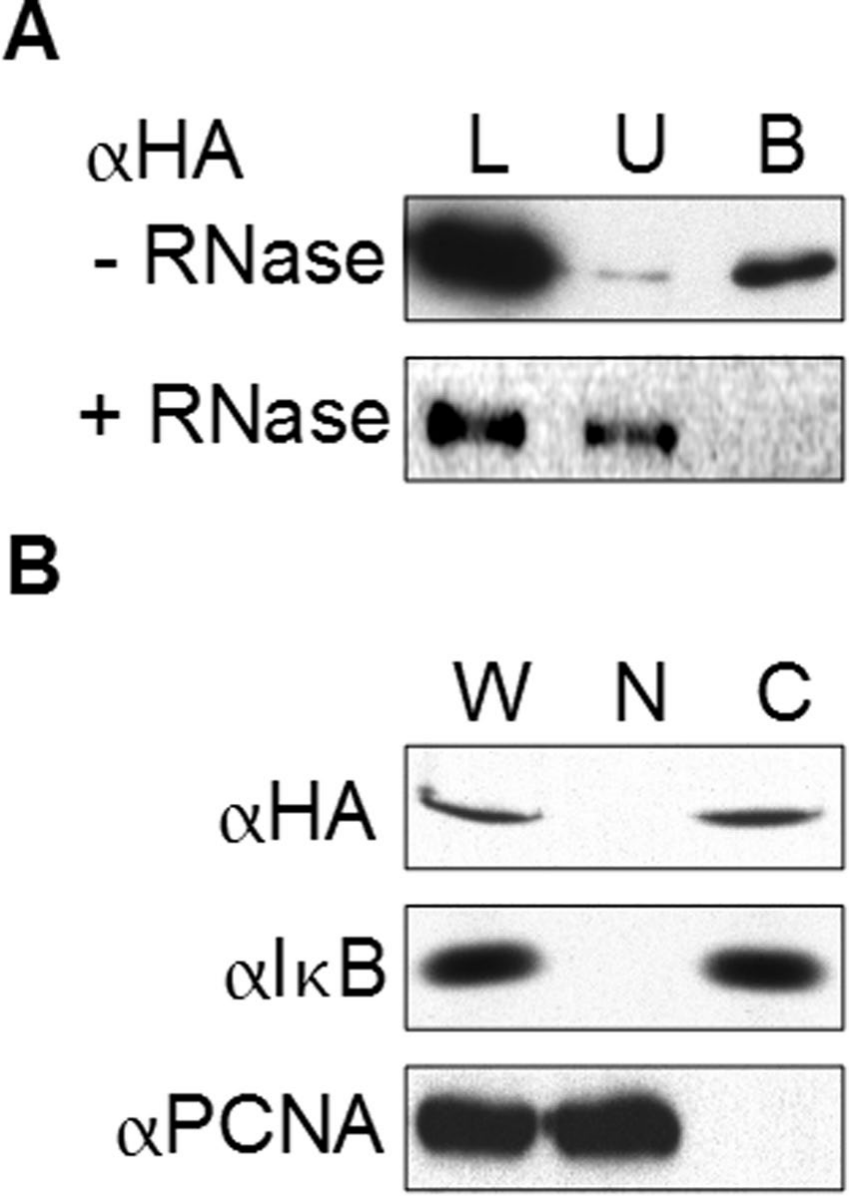
SpYY1 associates with mRNA and mRNPs in *Xenopus* oocytes. HA-SpYY1 was expressed in *Xenopus* oocytes via nuclear microinjection of a CMV-promoter-driven plasmid construct. Following 16 h of expression, oocytes were subjected to analysis. (**A**) HA-SpYY1-expressing oocyte lysates prepared in the presence of RNase inhibitor (−RNase, upper panel) or treated with RNaseA/T1 mixture (+RNase, lower panel) were subjected to oligo-dT cellulose chromatography, and aliquots of the load-on lysate (L), unbound fraction (U), and the bound fraction (**B**) were analyzed by Western blotting using an anti-HA antibody. (**B**) HA-SpYY1-expressing oocytes were lysed to produce whole-cell (W) lysates, or manually enucleated to isolate nuclear (N) and cytoplasmic (**C**) fractions as indicated above the panels and analyzed by Western blotting with anti-HA, anti-IκB (cytoplasmic marker), or anti-PCNA (nuclear marker) antibodies as indicated to the left of the panels.

The association of YY1 with mRNP particles in the cytoplasm of *Xenopus* oocytes has been shown to be dependent on the RNA-binding activity of YY1^43^. Furthermore, YY1 in *Xenopus* oocytes is entirely cytoplasmic but localization is unaffected by association of YY1 with cytoplasmic mRNA^40,42,43^. Given the differences in mRNA association observed in sea urchin versus frog egg lysates, we wished to analyze the biochemical properties of SpYY1 expressed in *Xenopus* oocytes. Epitope-tagged sea urchin YY1 (HA-SpYY1) was expressed in *Xenopus* oocytes via nuclear microinjection of a CMV promoter-driven plasmid construct containing the SpYY1 coding sequence. Following expression, oocyte lysates were analyzed by oligo-dT cellulose chromatography (Fig. 8A). SpYY1 was retained on oligo-dT cellulose matrix and its appearance in the mRNP fraction was abolished by treatment with RNase A, indicating that SpYY1 can associate with poly-A+ mRNA in frog oocytes, as has been previously demonstrated for native *Xenopus* YY1. In further experiments, HA-SpYY1-expressing oocytes were separated into nuclear and cytoplasmic fractions by manual enucleation and the resulting fractions were analyzed by Western blotting to determine the subcellular localization of exogenously expressed SpYY1 (Fig. 8B). This analysis revealed that HA-SpYY1 was entirely localized to the cytoplasmic compartment of oocytes similar to native *Xenopus* YY1^40,42,43^.

## Discussion

YY1 of vertebrate origin has been shown to have a physiologically relevant RNA-binding activity, however it is unknown if this activity is evolutionarily conserved. In addition, the role of YY1 in embryonic development remains poorly understood. Given our previous extensive analysis of *Xenopus* YY1^40,42–44,53^, we sought to analyze the biochemical properties and subcellular localization of YY1 in the purple sea urchin *Strongylocentrotus purpuratus*, a major model organism in developmental biology. We have identified the YY1 homologue in *S. purpuratus* and named it *SpYY1*. Sequence and phylogenetic analysis of SpYY1 show that it is highly similar to YY1 of mammalian origin. Importantly, SpYY1 is nearly completely identical with human YY1 over the regions associated with RNA- and DNA-binding, transcriptional activation, and transcriptional repression. SpYY1 shows far greater similarity to vertebrate YY1 than to the *D. melanogaster* homologue *PHO* and hence our suggestion for the name SpYY1 to highlight its sequence similarity to the vertebrate protein. Phylogenetic analysis of the protein sequence is consistent with accepted evolutionary relationships between major phyla of organisms, with SpYY1 most closely resembling the putative YY1 homologue of *C. intestinalis*, a member of the hemicordata which form a clade with echinoderms^51,52^. Furthermore, SpYY1 shared a branch of the phylodendrogram occupied by YY1 of other deuterostomes, and separate from the cnidarian *H. magnapapillata* and the protostome *D. melanogaster*.

The expression of the mRNA product of the putative SpYY1 gene an unidentified DNA-binding activity with affinity for the YY1 consensus DNA sequence have previously been observed in *S. purpuratus* eggs and embryos^48,50^. Here, we verify the expression of the SpYY1 protein by cloning of the coding sequence from cDNA prepared from ova and furthermore demonstrate the detection of endogenous SpYY1 via western blotting with anti-*Xenopus* YY1 antibodies. *In vitro* expression, purification, and biochemical analysis of the SpYY1 protein revealed that it possesses sequence-specific DNA binding activity to the YY1 DNA consensus sequence. We found that SpYY1 had a high-affinity RNA-binding activity. SpYY1 was observed to bind strongly to U-rich, single-stranded RNA but not to C-rich RNA. These findings are consistent with the biochemical properties reported previously for YY1 of *Xenopus* and human origin^42–45^. In addition to the RNA and DNA binding activities observed *in vitro*, *in vivo* RNA and DNA binding activities were observed that were specifically competed by YY1 consensus DNA oligonucleotides, suggesting endogenous SpYY1 has biochemical properties similar or identical to those observed *in vitro*.

Preparation of nuclear and cytoplasmic extracts of ova followed by western blotting revealed that SpYY1 is localized to the cytoplasm, indicating that SpYY1 likely does not function as a transcription factor at this stage of sea urchin development. This observation is consistent with previous data indicating YY1 is cytoplasmic in the oocytes, ova, and early embryos of *Xenopus* and mice^37,40^. While we did not detect SpYY1 in the nucleus of ova and did not analyze the subcellular localization of SpYY1 in subsequent stages of embryonic development, previous work has detected transcriptional activity from the YY1 DNA consensus sequence in fertilized *S. purpuratus* eggs, suggesting the protein is present in the nucleus following fertilization^48,50^. To further probe the role of cytoplasmic YY1, we analyzed the association of SpYY1 with mRNA in the ovum. Unlike YY1 in *Xenopus* oocytes and ova, SpYY1 did not associate with polyadenylated mRNA in the ovum. These data suggest that the association of YY1 with mRNA may not be evolutionarily conserved or may be a feature of YY1 biology unique to vertebrates or perhaps to *Xenopus* specifically. Furthermore, we hypothesize the presence of YY1 in the cytoplasm may be a mechanism for sequestering YY1 from DNA to prevent it from acting as a transcription factor until required for later development. Interestingly, expression of SpYY1 in *Xenopus* oocytes resulted in the sea urchin protein associating with maternal mRNA. This data indicates that the lack of association of SpYY1 with mRNA is likely not due to structural or sequence differences between the sea urchin and frog proteins. Indeed, if association of YY1 with mRNA in *Xenopus* was due to non-specific interaction of YY1 with mRNA it would be expected that the SpYY1 protein should associate with cytoplasmic RNA in the sea urchin ovum. The observed difference suggests that association of YY1 with mRNA in *Xenopus* and/or the lack thereof in sea urchins is a physiologically relevant phenomenon possibly mediated by co-factors not present in the sea urchin. These findings indicate the need for further work to understand the significance of YY1-mRNA interaction in *Xenopus* and the lack thereof in *S. purpuratus*.

Taken together, our data demonstrate the existence and expression of the protein product of the *Strongylocentrotus purpuratus SpYY1* (*Sp-Pho)* gene sequence described previously. The protein shows high similarity to vertebrate YY1 in terms of sequence, biochemical properties, and nuclear exclusion during early embryonic development. As a major model system in developmental biology, the cloning and characterization of YY1 in *S. purpuratus* will contribute substantially to the understanding of the function of YY1 in development. Demonstration of a conserved RNA-binding activity in YY1 and data showing the sea urchin protein functions similar to endogenous YY1 in *Xenopus* oocytes further underlines the need for future work to elucidate the biological significance of this aspect of YY1.

## Materials and Methods

### Preparation of Strongylocentrotus purpuratus ovum extract

Adult *Strongylocentrotus purpuratus* were obtained from West Wind Sealab Supplies Inc. Victoria, British Columbia, Canada in mid-January and maintained in sea water at 4 °C. Females were induced to release eggs by injection of 0.5 mL of 0.5 M KCl into the coelom. Ova were washed extensively in ice-cold sea water and collected by gentle centrifugation (100 × g, 2 min) to remove as much excess sea water as possible. Ova were lysed with twenty strokes of a Dounce homogenizer in ten volumes of buffer (180 mM KCH3CO2, 40 mM KCl, 40 mM NaCl, 50 mM Tris-HCl (pH 7.6), 5 mM MgCl2, 1 mM PMSF, 2 mM DTT, 40 U/mL RNase Inhibitor (Fermentas Inc; Burlington, Ontario, Canada). Lysates were centrifuged (20,000 × g, 20 min, 4 °C) and supernatants transferred to fresh tubes, avoiding the upper yolky layer, and stored at −80 °C.

### Cloning, bacterial expression, and purification of Strongylocentrotus purpuratus YY1

Ovum total RNA was isolated by lysis of ova obtained as above in ten volumes RNA isolation reagent (38% v/v water-saturated phenol, 9.5% w/v guanidium thiocyanate, 3.0% w/v ammonium thiocyanate, 0.1 M sodium acetate (pH 5.0), 5% v/v glycerol). Following complete homogenization, samples were mixed with 0.2 volumes chloroform, and centrifuged at 15,000 × g for 15 min. Supernatants were subsequently extracted once with phenol/chloroform (1:1) and once with chloroform/isoamyl alcohol (19:1) and total RNA was recovered by addition of an equal volume of isopropanol. Reverse transcriptions were performed as follows: Total RNA (5 µg) was combined with 0.5 µg oligo-dT(18) primer (New England Biolabs Inc., Pickering, Ontario, Canada) in a volume of 10.5 µL and heated at 70 °C for 10 min then chilled on ice. Mixtures were then supplemented with 4 µL of 5X RT buffer (Fermentas Inc. Burlington, Ontario, Canada), 20 U RNase inhibitor (Fermentas Inc., Burlington, Ontario, Canada), 1 mM each of dA/T/G/CTP, and 1 µL (200 U) of H-minus MuLV reverse transcriptase (Fermentas Inc., Burlington, Ontario, Canada) and incubated at 42 °C for 1 h. Reactions were terminated by heating at 70 °C for 10 min then chilled on ice and diluted to 50 µL with nuclease-free water and stored at −20 °C. Primers for PCR were designed so as to amplify the coding region of the predicted *S. purpuratus* YY1 mRNA sequence (accession #XM_785095.4). The forward primer included a BglII restriction site and had sequence 5′-GCGCAGATCTGACGCCGATGAAGTTGACATT whilst the reverse primer included an EcoRI site and had sequence 5′-GCGCGAATTCTCACAAAGGCAACGTGACTAC. Polymerase chain reactions contained 10 µL 10X Pfu buffer (Fermentas Inc. Burlington, Ontario, Canada), 2.5 mM MgSO4, 2 µL of diluted cDNA prepared as above, 250 µM dA/T/G/CTP, 250 µM each of forward and reverse primer, and 10 U Pfu DNA polymerase (Fermentas Inc., Burlington, Ontario, Canada) in a final volume of 100 µL. Reactions were incubated at 95 °C for 5 min followed by 30 cycles of 95 °C for 45 sec/56 °C for 45 sec/72 °C for 2 min, followed by 10 min at 72 °C. PCR fragments were purified, digested with BglII/EcoRI, purified, then ligated in-frame into BglII/EcoRI digested pRSetB, transformed into E. coli DH5α, and sequenced, generating pRSetB-SpYY1. For expression in Xenopus oocytes, the *S. purpuratus* YY1 coding sequence was subcloned at the BglII/EcoRI sites in-frame into pHA3 under control of the CMV promoter to generate pHA3-SpYY1. For bacterial expression of *S. purpuratus* YY1, pRsetB-SpYY1 was transformed into BL21(DE3) pLysS E. coli and protein expression was induced by addition of 2 mM IPTG. Bacterial lysis, protein purification, and renaturation of *S. purpuratus* YY1 were performed exactly as described previously for YY1 of Xenopus laevis origin^51^. Purified and renatured *S. purpuratus* YY1 protein was adjusted to a concentration of 10 µM and stored at −80 °C in small aliquots until use. All experiments involving sea urchin ova were repeated at least three times using eggs from different individual females to avoid the possibility of observing artifacts due to genetic or physiological differences between individuals.

### Multiple sequence alignment

Multiple sequence alignments were performed with ClustalX version 2.1 using default parameters, except that each alignment step was iterated. Sequences compared were as follows: *Homo sapiens* YY1 (Accession NP_003394.1), *Mus musculus* YY1 (Accession NP_033563.2), *Xenopus laevis* YY1 (Accession CAA54777.1), *Danio rerio* YY1 (Accession AAH71351.1), *Strongylocentrotus purpuratus* YY1 (Accession XP_790188), *Saccoglossus kowalevskii* YY1 (Predicted, Accession XP_002732622.1), *Ciona intestinalis* Zinc finger protein (C2H2)-66 (zf(C2H2)-66) (Predicted, Accession XP_009862335.1), *Drosophila melanogaster* PHO (Accession NP_524630.1), *Hydra magnipapillata* YY1 (Predicted, Accession CDG71191). From the resulting alignment, a phylogenetic tree was constructed using the drawgram program from the PHYLIP version 3.69 software package using the default parameters.

### Electrophoretic mobility shift assays

DNA probes were prepared by end-fill labelling of a double-stranded oligonucleotide containing the YY1 consensus DNA binding site with MuLV reverse transcriptase, and subsequently purified, according to standard protocols^54^. YY1-element DNA probes had the sequence 5′-CTAGGCGCTCCCCGGCCATCTTGGCGGCTGGT. RNA probes were labelled via phosphorylation with γ-32P-ATP and T4 polynucleotide kinase (Fermentas Inc., Burlington, Ontario, Canada), and then purified, according to standard methods^54^. RNA probe had the sequence 5′-UUUUUUUUUUUUUUUUUUUU, referred to hereafter as U(20). In competition experiments reactions were supplemented with 1 pmol of either YY1 consensus DNA element (sequence above), YY1 mutant consensus DNA element (5′-ACTGGCGCTCCGCGATTATCTTGGCGGCTGGT), unlabelled U(20) RNA, or unlabelled C(20) RNA (5′-CCCCCCCCCCCCCCCCCCCC). Electrophoretic mobility shift assays with recombinant protein contained 0.5 µM recombinant *S. purpuratus* YY1 protein, 0.1 pmol labelled probe, 50 mM NaCl, 50 mM Tris-HCl (pH 7.5), 2 mM MgCl2, 1 mM DTT, and 5% v/v glycerol in a final volume of 10 µL. Reactions containing RNA probes were additionally supplemented with 1 U of RNase inhibitor (Fermentas Inc., Burlington, Ontario, Canada). EMSA reactions with *S. purpuratus* ova lysate contained either 0.5 µg poly-dIdC (Invitrogen) for reactions with DNA probe, or 0.5 µg yeast tRNA and 2 U RNase inhibitor for reactions with RNA probe; 5 µL lysate, and 0.1 pmol probe in a final volume of 20 µL. For EMSAs of lysates treated with RNase, 10 µL of 10 mg/mL RNase A (Fermentas Inc; Burlington, Ontario, Canada) was added to 1 mL of lysate and incubated for 1 hr at 4 °C. EMSA reactions were incubated at room temperature for 20 min, supplemented with 1 µL (or 2 µL as appropriate) of 10X EMSA loading dye (80% v/v glycerol. 0.1% w/v Bromophenol Blue, 0.1% w/v xylene cyanol FF), and immediately loaded on 5% polyacrylamide 0.5X TBE (44 mM Tris base, 44 mM boric acid, 0.5 mM EDTA, (pH 8.3)) non-denaturing gels and electrophoresed for 2.5 h at 150 V. Gels were then dried and autoradiographed by exposure to XB-1 film (Kodak, Rochester, New York, USA) overnight at −80 °C with an intensifying screen. All experiments involving studies of recombinant protein were replicated a minimum of three times.

### Isolation of *S. purpuratus* messenger ribonucleoprotein particles

Ova lysates (1 mL), either untreated or treated with RNase as above, were applied to 100 mg of oligo-dT cellulose type VII (New England Biolabs) for 2 h at 4 °C with gentle agitation. Following incubation, matrices were collected by centrifugation (1000 × g, 5 min, 4 °C) and washed three times with 1 mL of ice-cold buffer (180 mM KCH3CO2, 40 mM KCl, 40 mM NaCl, 50 mM Tris-HCl (pH 7.6), 5 mM MgCl2, 2 mM DTT). Bound messenger ribonucleoprotein complexes were eluted by heating matrices at 95 °C with 250 µL of 1X SDS-PAGE loading buffer (50 mM Tris-HCl pH 6.8, 5% v/v glycerol, 2% w/v sodium dodecyl sulfate, 50 mM 2-mercaptoethanol, 0.05% w/v Bromophenol Blue).

### Isolation of nuclei from *S. purpuratus* oocytes

Nuclei from oocytes were isolated according to the method of Thaler *et al*.^53^. Briefly, unfertilized eggs were collected as above by injection of potassium chloride solution and washed in ice-cold sea water. Following homogenization and initial centrifugation to collect nuclei the supernatant containing the cytoplasmic fraction was combined with 1/10 volume of 100% w/v trichloroacetic acid, and precipitated protein collected by centrifugation (20,000 × G, 20 min, 5 °C). The resulting pellet was washed 3X 1 mL in ice-cold 80% acetone, dried, resuspended in ten pellet volumes of 1X SDS-PAGE loading buffer, heated at 95 °C for 5 min, centrifuged to remove debris, and stored at −20 °C. The remaining pellet of impure nuclei from the initial homogenization step was then processed exactly as described by Thaler *et al*. except that the final isolated nuclei were lysed directly in ten volumes 1X SDS-PAGE loading buffer, heated at 95 °C for 5 min, centrifuged to remove debris, and stored at −20 °C.

### Expression and Analysis of *S. purpuratus* YY1 in Xenopus laevis Oocytes

All experiments involving *Xenopus laevis* frogs were carried out according to The Guide for the Care and Use of Laboratory Animals and were approved by the Ethics Committee of the University of Saskatchewan. Oocytes were isolated by collagenase treatment of ovarian tissue surgically removed from adult female *Xenopus laevis* according to established procedures^54^ and maintained at 18 °C in OR2 buffer (82.5 mM NaCl, 2.5 mM KCl, 1 mM CaCl2, 1 mM MgCl2, 1 mM Na2HPO4, 5 mM HEPES, 3.8 mM NaOH (Final pH 7.8)). Stage VI oocytes were selected according to the criteria of Dumont^55^. Microinjections were performed using a Narashige(R) model IM-300 microinjector. Expression of HA-tagged *S. purpuratus* YY1 in oocytes was achieved by nuclear microinjection of 20 ng of plasmid in 20 nL of 0.1 M KCl at a pressure of 12 psi. Typical injection rates at the indicated pressure were 0.1 nL/msec ± 20%. Following incubation for 16 h at 18 °C for expression of HA-SpYY1 protein, oocytes were lysed in 10 volumes mRNP buffer (150 mM NaCl, 50 mM Tris-HCl (pH 7.5), 2 mM MgCl2, 10% v/v glycerol, 1 mM DTT, 1X Protease inhibitor cocktail (Sigma), 100 U/mL RNase inhibitor (Fermentas Inc., Burlington, Ontario, Canada)), with the exception that RNase inhibitor was omitted for lysates intended for subsequent RNase treatment. Lysates were centrifuged at 15,000 × g for 12 min at 4 °C. Supernatants were removed and extracted with an equal volume of 1,1,2-trifluorotrichloroethane to remove yolk proteins and clarified supernatants were stored at −80 °C until use. Where indicated, lysates were treated with 10 µL per mL lysate of RNase A/T1 mix (Fermentas Inc. Burlington, Ontario, Canada) at room temperature for 30 min. For oligo-dT cellulose chromatography; lysate (500 µL) was applied to 100 µL type VII oligo-dT cellulose (New England Biolabs Inc., Pickering, Ontario, Canada) followed by incubation for 2 h at 4 °C with gentle rotation. Matrices were collected by centrifugation at 1000 × g for 1 min, and washed three times with 1 mL of mRNP buffer (see above), then eluted by heating to 95 °C for 5 min with 200 µL of 1X SDS-PAGE loading buffer. For nuclear and cytoplasmic lysates, HA-SpYY1 expressing oocytes were manually enucleated and separated nuclei and cytoplasms were each lysed in mRNP buffer containing protease inhibitor but lacking RNase inhibitor. Protein concentrations of nuclear and cytoplasmic extracts were determined by the method of Bradford^56^.

### Western Blotting and Antibodies

#### Sodium dodecyl sulfate

polyacrylamide gel electrophoresis (SDS-PAGE) was carried out on 10% polyacrylamide gels according to established methods^57^ and separated proteins were transferred to PVDF membranes or gels were stained with Coomassie brilliant blue according to standard procedures^52^. *S. purpuratus* YY1 was detected using a custom-made polyclonal antibody raised in rabbits against a synthetic peptide with sequence AMRKHLHTHGPRVH corresponding to amino acids 268–281 of *Xenopus laevis* YY1 (Accession #NP_001087404), described previously^51^, and used at a concentration of 1:5000. Other antibodies used were as follows: Rabbit anti-HIS6 (Santa Cruz Biotechnology Inc., Santa Cruz, CA, USA, Cat. #SC-803) at 1:5000; mouse anti-HA (Santa Cruz Biotechnology Inc., Santa Cruz, CA, USA, Cat. #SC-7392) at 1:5000; rabbit anti-IκB-α (Santa Cruz Biotechnology Inc., Santa Cruz, CA, USA, Cat. #SC-847) at 1:1000; mouse anti-PCNA (Santa Cruz Biotechnology Inc., Santa Cruz, CA, USA, Cat. #SC-56) at 1:5000; goat anti-Histone H3 (Santa Cruz Biotechnology Inc., Santa Cruz, CA, USA, Cat. #SC-8654) at 1:1000, goat anti-beta-actin (Santa Cruz Biotechnology Inc., Santa Cruz, CA, USA, Cat. #SC-1615) at 1:1000, goat anti-rabbit HRP conjugate (Bio-Rad Inc., Mississauga, Ontario, Canada, Cat. #170–6515) at 1:5000; mouse anti-goat HRP conjugate (Santa Cruz Biotechnology Inc., Santa Cruz, CA, USA, Cat. #SC-2354) at 1:5000, and goat anti-mouse HRP conjugate (Bio-Rad Inc., Mississauga, Ontario, Canada, Cat. #170-6516) at 1:5000. Following incubation with primary and secondary antibodies and washing, blots were incubated in chemiluminescence reagent (100 mM Tris-HCl (pH 8.5), 0.2 mM p-coumaric acid, 1.25 mM luminol, 0.01% v/v hydrogen peroxide) and exposed to XB-1 film (Kodak, Rochester, NY, USA).
